# The Oxysterol 7-Ketocholesterol Reduces Zika Virus Titers in Vero Cells and Human Neurons

**DOI:** 10.3390/v11010020

**Published:** 2018-12-30

**Authors:** Katherine A. Willard, Christina L. Elling, Steven L. Stice, Melinda A. Brindley

**Affiliations:** 1 Department of Infectious Diseases, College of Veterinary Medicine, University of Georgia, Athens, GA 30602, USA; katherine.willard@duke.edu; 2 Department of Animal and Dairy Science, Regenerative Bioscience Center, College of Agriculture and Environmental Science, University of Georgia, Athens, GA 30602, USA; christina.elling@ucdenver.edu (C.L.E.); sstice@uga.edu (S.L.S.); 3 Department of Infectious Diseases, Department of Population Health, Center for Vaccines and Immunology, College of Veterinary Medicine, University of Georgia, Athens, GA 30602, USA

**Keywords:** Zika virus, antiviral compounds, neural cells, viral replication

## Abstract

Zika virus (ZIKV) is an emerging flavivirus responsible for a major epidemic in the Americas beginning in 2015. ZIKV associated with maternal infection can lead to neurological disorders in newborns, including microcephaly. Although there is an abundance of research examining the neurotropism of ZIKV, we still do not completely understand the mechanism by which ZIKV targets neural cells or how to limit neural cell infection. Recent research suggests that flaviviruses, including ZIKV, may hijack the cellular autophagy pathway to benefit their replication. Therefore, we hypothesized that ZIKV replication would be impacted when infected cells were treated with compounds that target the autophagy pathway. We screened a library of 94 compounds known to affect autophagy in both mammalian and insect cell lines. A subset of compounds that inhibited ZIKV replication without affecting cellular viability were tested for their ability to limit ZIKV replication in human neurons. From this second screen, we identified one compound, 7-ketocholesterol (7-KC), which inhibited ZIKV replication in neurons without significantly affecting neuron viability. Interestingly, 7-KC induces autophagy, which would be hypothesized to increase ZIKV replication, yet it decreased virus production. Time-of-addition experiments suggest 7-KC inhibits ZIKV replication late in the replication cycle. While 7-KC did not inhibit RNA replication, it decreased the number of particles in the supernatant and the relative infectivity of the released particles, suggesting it interferes with particle budding, release from the host cell, and particle integrity.

## 1. Introduction

Zika virus (ZIKV) is an emerging arbovirus that gained public attention in 2015 when maternal infection in the Americas was causally associated with congenital birth defects, including microcephaly [1]. ZIKV belongs to the *Flaviviridae* family and is related to other important human pathogens, including dengue (DENV), yellow fever (YFV) and West Nile (WNV). ZIKV is an enveloped virus with a positive-sense RNA genome that translates into a single polypeptide, which is later cleaved into three structural and seven nonstructural viral proteins. Upon binding to host cell receptors, the cell engulfs virions through clathrin-mediated endocytosis [2]. Low pH in the endosome triggers viral-cellular membrane fusion, releasing the viral RNA genome into the host cell cytoplasm [2]. Transcription occurs in the cytoplasm and translation of ZIKV proteins occurs on membrane scaffolds near the endoplasmic reticulum (ER) [3].

Autophagy is a normal cellular process used to recycle cytoplasmic components in eukaryotic cells. The autophagy pathway is activated by mTOR [4]. This activation signals the production of lipid membranes that engulf targeted cytoplasmic components, forming autophagosome vesicles. Eventually, the autophagosomes fuse with lysosomes to form autophagolysosomes, which degrade cargo and prepare it to either be recycled or ejected from the cell [4]. Because cells always contain components that need to be recycled, the autophagy pathway is constantly on at a basal level. Different stimuli or stresses, such as pathogen infection, can alter basal levels of autophagy. For example, selectively encasing intercellular bacteria and targeting them for autophagic degradation is part of the innate immune response pathway for dealing with *Salmonella enterica* serovar Typhimurium and *Mycobacterium tuberculosis* [5,6].

While the host can utilize this pathway to rid itself of some pathogens, many flaviviruses, including Dengue, Hepatitis C, and Zika viruses, hijack this process to benefit their own replication [4,7,8,9]. The autophagy process mobilizes cellular membranes. Flaviviruses replicate on membranes and appear to benefit from initiating early cellular autophagy processes [7,10]. Chemical inducers of autophagy, such as rapamycin, slightly increase levels of viral RNA and infectious particle production [11,12,13]. In addition, chemical inhibitors of autophagy decrease particle production [12,13]. Some autophagy inhibitors, such as bafilomycin A, prevent the acidification of autophagolysosomes. Such compounds do not selectively block acidification of only autophagolysosomes, but also alter the pH of other endosomal vesicles. Because flavivirus entry requires an acidic endosome environment to trigger membrane fusion, some of the drugs may be inhibiting initial entry. Therefore, their effects on autophagy may be unrelated to the flavivirus inhibition. Flavivirus replication appears to be enhanced when the autophagy pathway is started, but is stalled and autolysosome degradation is blocked [4].

Autophagy also affects other aspects of cell biology that may influence viral pathogenesis, including induction of the interferon response [14]. However, depending on the location and timing of infection, autophagy can also be antiviral. For instance, experiments in *Drosophila* indicate that ZIKV infection in the brain induces an NF-κB/dSTING (*Drosophila* stimulator of interferon genes) signaling pathway, which induces autophagy and protects against ZIKV infection [15]. Therefore, autophagy can be very consequential to viral replication and may play a role in ZIKV pathogenesis [4].

Since autophagy and ZIKV replication are intertwined, small molecules that induce or inhibit stages of the autophagy pathway may alter ZIKV production and spread in host cells. To elucidate these interactions, we screened a library of 94 autophagy inducers or inhibitors in Vero and C6/36 cells infected with ZIKV. Surprisingly, only about 30% of compounds reduced ZIKV titer by at least one log compared to control. We performed subsequent experiments in both Vero cells and human neurons with the compounds that reduce ZIKV replication without inhibiting cell viability. We identified one compound, 7-ketocholesterol (7-KC), which effectively reduced ZIKV titer in human neurons without affecting cellular viability. 7-KC blocked late stages of ZIKV replication, suggesting it reduces particle integrity and budding efficiency from host cells.

## 2. Materials and Methods

### 2.1. Cell Lines

Vero cells were maintained in DMEM with 5% fetal bovine serum (FBS) at 37 °C, 5% CO_2_. C6/36 *Ae. albopictus* cells (ATCC CRL-1660) were maintained in L-15 Leibovitz Medium with l-glutamine and 10% FBS at 28 °C. Human neurons were made by differentiating hNP1 cells and were obtained from ArunA Biomedical, Inc. [16]. Neurons were maintained in medium containing AB2^TM^ basal medium supplemented with ANS^TM^ neural supplement (both from ArunA Biomedical Inc. Athens, GA, USA), 2 mM l-glutamine (Gibco, ThermoFisher, Waltham, MA, USA), 2 U/mL penicillin (Gibco, ThermoFisher, Waltham, MA, USA), 2 μg/mL streptomycin (Gibco, ThermoFisher, Waltham, MA, USA), and 10 ng/mL leukemia inhibitory factor (LIF) (Millipore, Billerica, MA, USA) and plated on cell culture dishes coated with Matrigel 1:100 (B&D, Franklin Lakes, NJ, USA).

### 2.2. Virus Isolates

ZIKV SPH is a low-passage (<5), Asian-lineage isolate derived from an infected patient in Brazil in 2015 and obtained from the Oswaldo Cruz Foundation (FIOCRUZ). ZIKV IbH 30656 (ATCC # VR-182) is a highly-passaged, African-lineage isolate derived from a human patient in Nigeria in 1968. Following its initial isolation, IbH was extensively passaged in mouse brains.

### 2.3. Reagents

We used the Enzo Screen-Well Autophagy Library for these experiments (category number: BML-2837-0100; Lot number: 03181609B; Version 1.5). The compounds came resuspended at 10 mM in dimethyl sulfoxide (DMSO). To prevent DMSO related toxicity, we diluted the stocks 1:1000 and screened the compounds at a final concentration of 10 μM. DMSO was included in all of our untreated controls.

### 2.4. Autophagy Compound Library Screen

Vero and C6/36 cells were plated at densities of 1.5 × 10^5^ cells/well and 2 × 10^5^ cells/well, respectively in 24-well plates. The cells were incubated for 2–4 h and then treated with the autophagy compounds at a final concentration of 10 µM. Immediately after treatment, the cells were infected with ZIKV (MOI 0.1). Vero cells were incubated for 40 h and the C6/36 cells for 96 h, then supernatants were collected and titrated by determining the 50% tissue culture infective dose (TCID_50_) according to the Spearman-Karber method [17,18]. Results from treated cells were compared to results from DMSO controls.

### 2.5. Autophagy Detection

Vero cells were plated at densities of 3 × 10^5^ cells/well in 12-well plates. The cells were incubated for 2–4 h and then treated with the autophagy compounds at a final concentration of 10 µM. Twenty-four hours following treatment, the cells were lifted in trypsin, washed in PBS, and stained with the CYTO-ID Autophagy Detection Kit 2.0 (Enzo, Farmington, NY, USA). The autophagy activity factor (AAF) [19] was determined by comparing the green mean fluorescence intensities (MFI) of treated and DMSO control cells using the formula:

$$\mathrm{AAF}=100\times \left(\frac{\mathrm{MFItreated}-\mathrm{MFIcontrol}}{\mathrm{MFItreated}}\right)$$

### 2.6. Cell Viability

Vero and C6/36 cells were plated at densities of 1.5 × 10^4^ cells/well and 2 × 10^4^ cells/well, respectively in 96-well plates. The cells were incubated for 2–4 h and then treated with the autophagy compounds at a final concentration of 10 µM. Vero cells were incubated for 40 h and the C6/36 cells incubated for 96 h. Metabolic activity (ATP abundance), which correlates to cell viability, was determined with CellTiter-Glo Luminescent Cell Viability Assay (Promega, Madison, WI, USA) and a Glomax plate reader per the manufacturer’s instructions. The experiment was repeated at least three independent times. Results from treated cells were compared to results from DMSO controls.

### 2.7. Human Neuron Response to Autophagy Compounds

The human PSC line WA09 (WiCell 0062, Madison, WI, USA) was used to derive human neural progenitor cells (hNP1TM 00001) as previously described [16]. ArunA Biomedical, Inc. (Athens, GA, USA) differentiated the hNP1 cells into neurons and we cultured the neurons as previously described [20]. Before plating neurons, each well was coated with Matrigel (1:100 dilution; (B&D, Franklin Lakes, NJ, USA) and incubated for at least 30 min at room temperature. After incubation, the Matrigel was removed and the wells were washed twice with PBS. Neurons were immediately plated at densities of 2 × 10^5^ cells/well in 24-well plates and 3 × 10^4^ cells/well in 96-well plates. The following day cells were treated with 10 µM final concentration of compounds, and the 24-well plate was infected with ZIKV SPH (MOI 1.0). The infected/compound-treated neurons were incubated for 4 days then the supernatants were collected and neuron viability was determined as described above. The compound-treated supernatants were titrated via TCID_50_.

### 2.8. 7-Ketocholesterol Dose-Response

Vero cells were plated at 1.5 × 10^5^ cells/well in a 24-well plate and 1.5 × 10^4^ cells/well in a 96-well plate and incubated for 16 h. Cells were treated with the indicated concentration of 7-KC or DMSO control. The cells in the 24-well plate were immediately infected with MOI 1 of ZIKV SPH. After 2 h, the infectious media was removed and replaced with the same concentrations of 7-KC treated media as indicated. The cell supernatants were collected 24 h following infection and titers were reported in TCID_50_ units. ZIKV titers from 7-KC treated cells were compared to those from DMSO-treated controls.

### 2.9. 7-Ketocholesterol Time-of-Addition

Vero cells at a density of 3 × 10^5^ cells/well in 24-well plates were treated with either 10 µM of 7-KC or 30 mM of ammonium chloride under the following conditions: 2 h prior to ZIKV infection, at the same time as ZIKV infection, 2 h following ZIKV infection, or 6 h following ZIKV infection. Regardless of treatment condition, the cells were infected with ZIKV SPH MOI 0.1 for 2 h. After 2 h, the infectious media was removed and replaced with fresh media. For the cells treated at the same time as infection, the fresh media was re-treated with the compounds at the same concentrations as above. The supernatants were collected at 24 hours post infection (hpi) and titers were reported in TCID_50_ units. ZIKV titers from treated cells were compared to those from controls, which were treated with either DMSO or DMEM at the time of infection and retreated after the media change.

### 2.10. 7-Ketocholesterol and ZIKV Pre-Incubation

Vero cells were plated at 1.5 × 10^5^ cells/well in a 24-well plate and incubated for 18 h. The next day, ZIKV stock was pre-incubated with 200 µM of 7-KC for 1 h at room temperature. After incubation, the ZIKV/7-KC mixture was diluted and added to the Vero cells. The dilution resulted in an infection at MOI 0.1 and final concentration of 7-KC was approximately 0.2 µM (below the active range of 7-KC as determined by our dose-response curve). The cell supernatants were collected at 40 hpi and titers were determined. Pre-incubation results were compared to DMSO controls.

### 2.11. qRT-PCR

Vero cells were infected with ZIKV SPH and treated with compounds in the same manner as the time-of-addition experiment. Supernatants and cellular lysates were collected at 24 hpi. Viral RNA was isolated using the ZR Viral RNA kit (Zymo, Irvine, CA, USA) and cellular RNA was isolated with the RNAeasy Mini kit (Qiagen, Hilden, Germany). Both total and viral RNA samples were reverse-transcribed (RT) to cDNA (High Capacity RNA-to-cDNA Kit, Applied Biosystems, Foster City, CA, USA). To quantify the copies of ZIKV genomes we used the cDNA in a quantitative PCR (qPCR) reaction assay using TaqMan Gene Expression Master Mix (Applied Biosystems, ThermoFisher, Waltham, MA, USA), primers and probes (F: ZIKV 1086, R: ZIKV 1162c, ZIKV 1107-FAM; TaqMan MGB Probe; Invitrogen Custom Primers) [21]. Each sample was analyzed in duplicate, and each plate contained a DNA plasmid standard curve (ZIKV molecular clone), no template, and no primer controls. ZIKV copy numbers were extrapolated from the generated standard curve using the Applied Biosystems protocol. The limit of detection was experimentally established to be 30 copies (10^−16^ g). Final copy numbers were adjusted by back-calculations to the total RNA and cDNA volume and expressed as copies per sample.

### 2.12. Chemical Structures

We created chemical structures using PyMOL version 2.1.1. Structure files were downloaded from Pubchem (https://pubchem.ncbi.nlm.nih.gov/).

### 2.13. Statistics

All experiments were performed in at least three independent trials. We assessed variation across trials using the standard error of the mean. We used the student’s unpaired *t*-test to test for significance when comparing data plotted on a linear scale and Welch’s *t*-test of unequal variance when data is plotted on a log-scale. Significance values are: * *p* < 0.05; ** *p* < 0.01; *** *p* < 0.001; NS- not significant.

## 3. Results

### 3.1. Autophagy Compound Screen in Vero Cells

**Figure 1:**
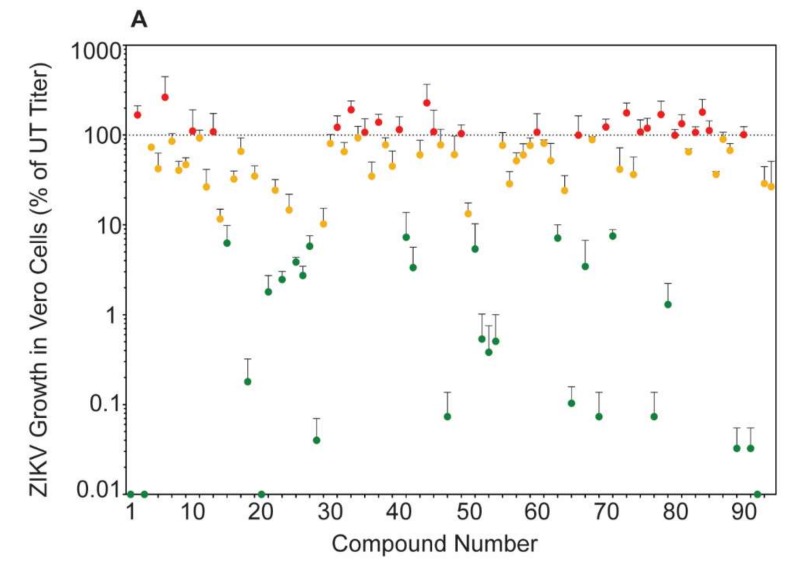

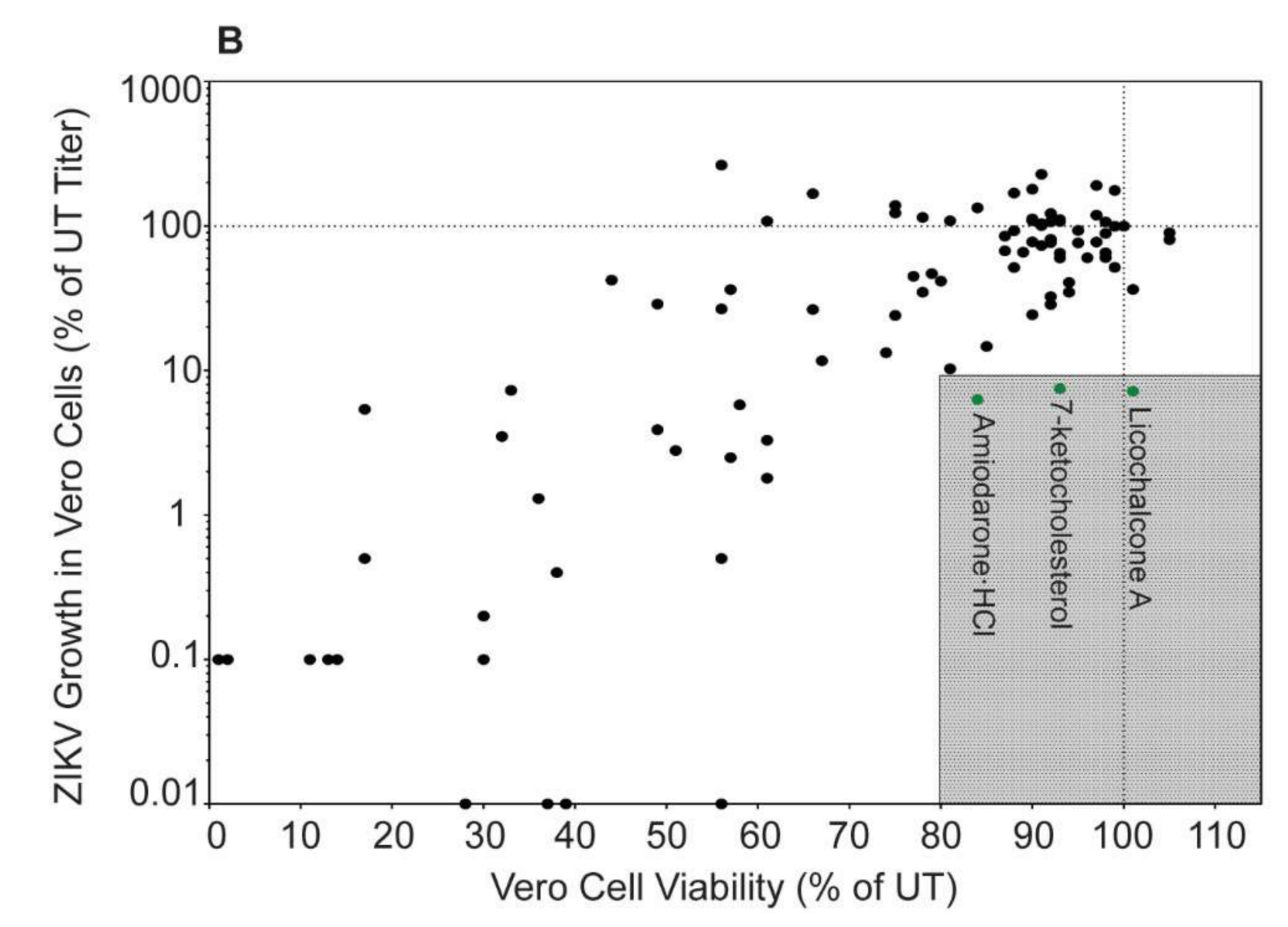
Autophagy compound screen in Vero cells. (**A**) ZIKV SPH growth in compound-treated Vero cells as a percent of ZIKV titer in untreated Vero cells. Red symbols represent compounds that increased ZIKV titer compared to the untreated control, yellow symbols represent compounds that reduced titer between 10 and 99% of control, and green symbols represent compounds that reduced ZIKV titer below 10% of control (at least 1 log of viral growth). Error bars represent the standard error of the mean (SEM) from three independent trials. The compounds are numbered according to Table 1. (**B**) ZIKV growth in Vero cells versus viability of compound-treated Vero cells. The shaded box represents the zone in which ZIKV titer is reduced to 10% or lower than the untreated control and cell viability is at least 80% of control. Compounds that meet these criteria are in green and labeled with their names.

We tested the ability of the Enzo Screen-Well Autophagy Library (94 compounds listed in Table 1) to reduce ZIKV titer in mid-logarithmic growth phase (40 h post infection) [22], without negatively impacting viability compared to controls in Vero cells. These compounds produced a wide range of effects on ZIKV titer, though only approximately 30% were able to decrease ZIKV titer by at least one log compared to control (Figure 1A). Similar to previously published data, treatment with the prototypical autophagy inducer rapamycin increased ZIKV titers whereas the prototypical autophagy inhibitor hydroxychloroquine decreased titers [11,12,13]. Of the 94 compounds, three (amiodarone-HCl, 7-ketocholesterol, and licochalcone A) reduced ZIKV titer to below 10% of control while maintaining cell viability above 80% of control (Figure 1B).

Amiodarone-HCl, 7-ketocholesterol, and licochalcone A are all characterized as autophagy inducers. Based on previous work, autophagy inducers would be predicted to increase ZIKV titers. However, amiodarone-HCl, 7-ketocholesterol, and licochalcone A decreased ZIKV titers in our assay. To confirm that these compounds induced autophagy in the Vero cells, we stained treated cells with CYTO-ID, a dye that binds to autophagic vesicles [19]. We detected increased fluorescence when cells were treated with rapamycin as well as with amiodarone-HCl, 7-ketocholesterol, and licochalcone A, confirming the compounds induce autophagy in Vero cells (Table 2). Amiodarone-HCl treatment in particular led to a very large increase in fluorescence intensity. While rapamycin and licochalcone A both induce autophagy through mTOR signaling, amiodarone-HCl induces autophagy through mTOR independent pathways [23].

### 3.2. Autophagy Compound Screen in Vero Cells Infected with ZIKV IbH

**Figure 2:**
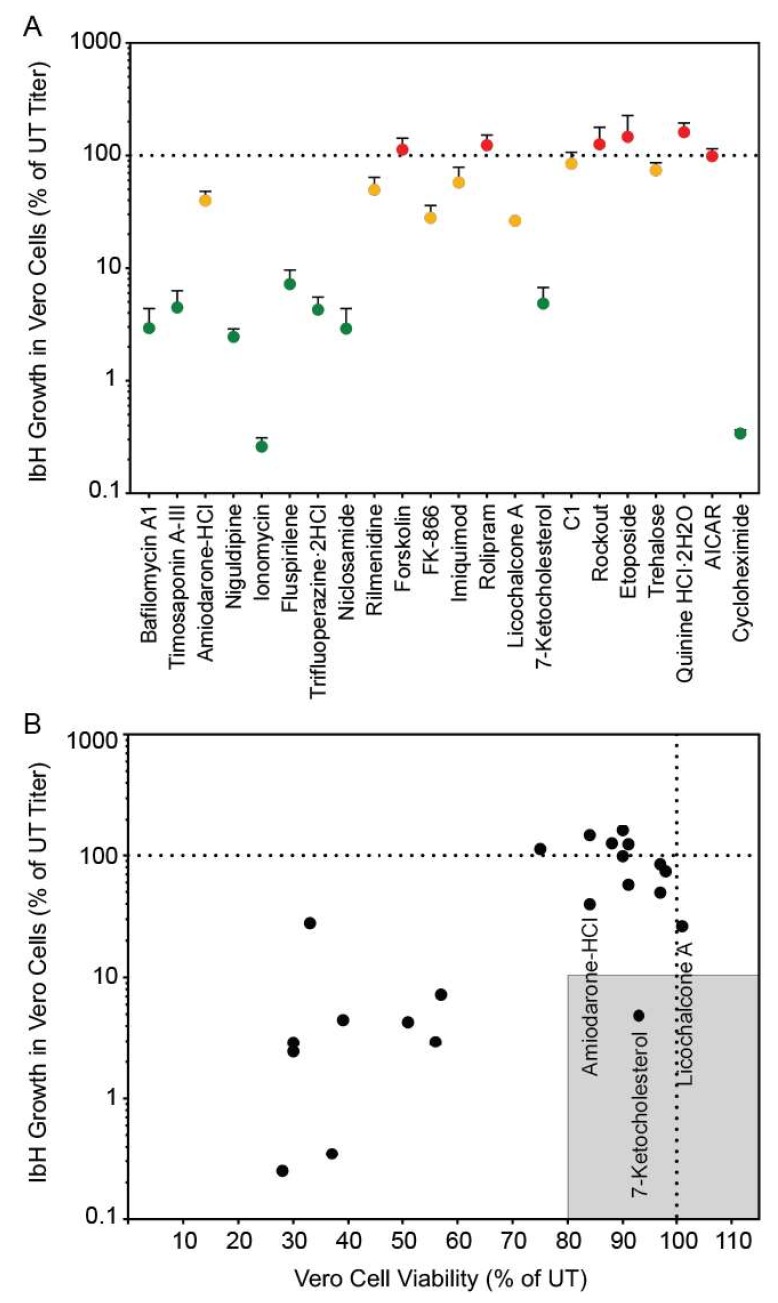
African- lineage ZIKV IbH growth in compound-treated Vero cells as a percent of ZIKV titer in untreated Vero cells (**A**). The color-codes are the same as in Figure 1A. (**B**) ZIKV IbH growth in Vero cells versus viability of compound-treated Vero cells. The shaded box represents the zone in which ZIKV titer is reduced to 10% or lower than the untreated control and cell viability is at least 80% of control.

There are two genetic lineages of ZIKV [24], and isolates from these different lineages can produce distinct phenotypes in infected cells [20,22,25,26]. Therefore, we tested the ability of a subset of 22 autophagy compounds to inhibit African lineage ZIKV IbH in Vero cells (Figure 2). We chose this subset based on our results with SPH; one third of the compounds decreased SPH titer below 10%, one third decreased SPH titer between 10 and 99%, and the final one third increased SPH titer compared to control. These compounds affected African-lineage IbH replication in a manner similar to the effects on ZIKV-SPH (Figure S1). Thus, the compounds effect on ZIKV replication is not lineage specific. While amiodarone-HCl, 7-ketocholesterol, and licochalcone A all reduced ZIKV IbH titers, only 7-KC dropped titers more than a log compared to control. Because we did not identify major differences in ZIKV titer across lineages, we continued with Asian-lineage ZIKV SPH for the remainder of our experiments.

### 3.3. Autophagy Compound Screen in C6/36 Cells

**Figure 3:**
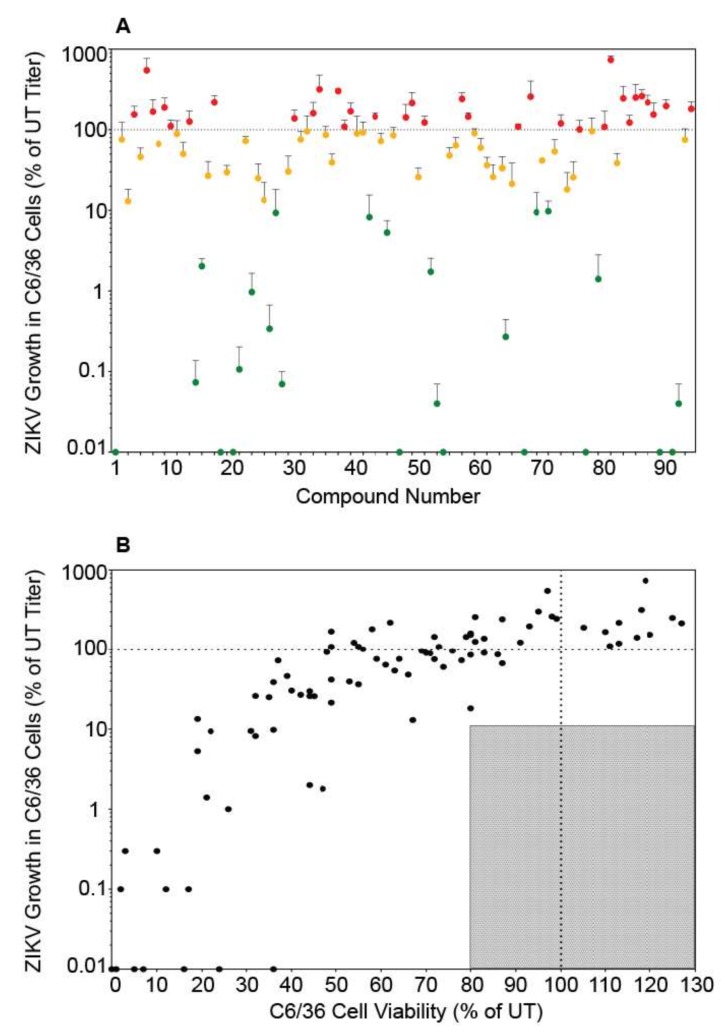
Autophagy compound screen in C6/36 cells. (**A**) ZIKV growth in compound-treated C6/36 cells as a percent of ZIKV titer in untreated C6/36 cells. The labels and color-codes are the same as in Figure 1A. (**B**) ZIKV growth in C6/36 cells versus viability of compound-treated C6/36 cells. As in Figure 1, the shaded box represents the zone in which ZIKV titer is reduced to 10% or lower than the untreated control and cell viability is at least 80% of control. No compounds fit these criteria in C6/36 cells.

ZIKV is transmitted primarily by *Aedes* mosquitoes and the virus must infect and replicate in mosquito cells to be transmitted. To determine if the autophagy compound panel altered ZIKV replication in mosquito cells in a similar manner to mammalian cells, we performed the screen in C6/36 *Aedes albopictus* cells. Because mosquito cells have a slower metabolic rate, cellular viability and viral titers were examined 96 h post-infection/treatment [22]. Once again, the autophagy compounds produced a wide range of effects on SPH titer (Figure 3A). However, unlike in Vero cells, we did not identify any compounds that reduced ZIKV titer to below 10% of control while maintaining viability above 80% of control (Figure 3B). Some compounds did not produce the expected results. For example, rapamycin which increase particle production in Vero cells, slightly decreased titers in C6/36 cells. Interestingly, more compounds increased SPH titer in C6/36 cells compared to Vero cells (Figure 2A and Figure 3A, Figure S2, Table 1). While 27 compounds in Vero cells produced virus >90% of control, 38 compounds behaved similarly in C6/36 cells. The insect and mammalian autophagy pathways are highly conserved [27] and many of the autophagy altering compounds affected ZIKV replication in Vero and C6/36 cells in a similar fashion (Figure S2). However we found opposing effects with some compounds. Cell type specific cytotoxicity may explain many of the differences observed. For example, brefeldin A was toxic in Vero cells, resulting in little virus production, yet brefeldin A did not alter viral titers or cell viability in C6/36 cells. While amiodarone HCl, licochalcone A, and 7-ketocholesterol (7-KC) inhibited ZIKV replication in both Vero and C6/36 cells, they were more toxic to the C6/36 cells. Many compounds resulted in significant cell death in the C6/36 cells and may be related to the increased time period the cells were exposed to the compounds. We did not identify any compounds that inhibited viral replication without significantly affecting cell viability.

### 3.4. Autophagy Compound Screen in Human Neurons

**Figure 4:**
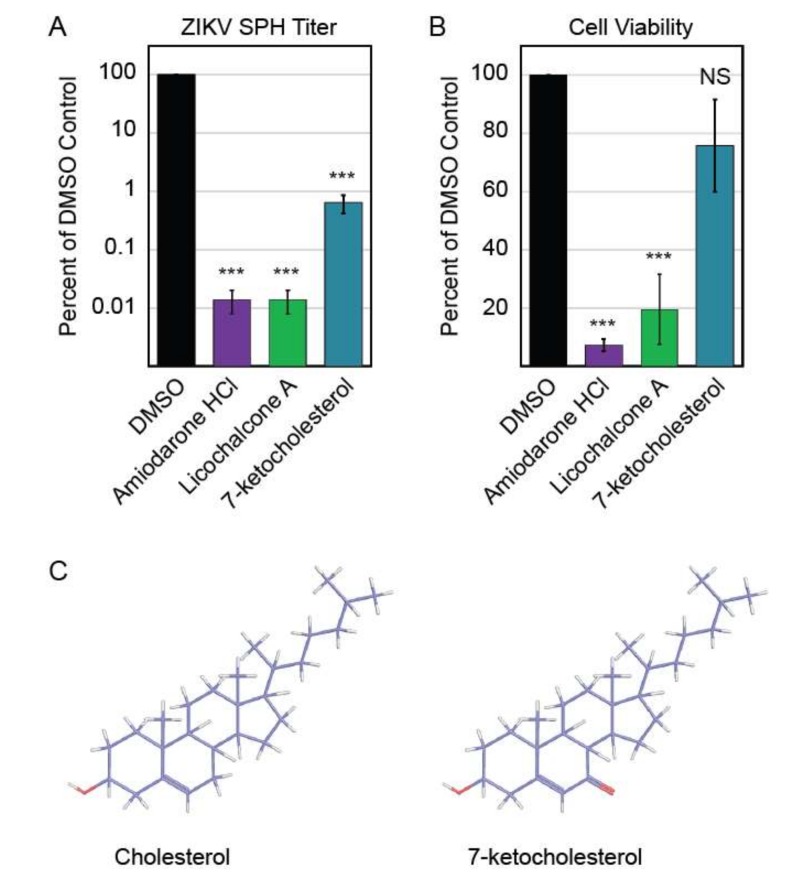
Autophagy compound screen in human neurons. (**A**) Impact of autophagy compounds on ZIKV replication in neurons and (**B**) neuron viability. (**C**) Chemical structure of cholesterol (PubChem CID 5997) versus 7-ketocholesterol (PubChem CID 91474). Carbon atoms are shaded in purple, hydrogen atoms are shaded in grey, and oxygen atoms are shaded in red.

The three compounds that reduced ZIKV titer without impacting Vero cell viability were further characterized to determine these compounds’ abilities to inhibit ZIKV replication in human neurons, a cell line more relevant to clinical ZIKV infection. We performed this screen with neurons derived from human neural progenitor cells, which have been characterized in previous ZIKV experiments [20]. Of these three compounds, only 7-ketocholesterol (7-KC) reduced ZIKV titer without significantly decreasing neuron viability (Figure 4A,B). Structurally, 7-KC is very similar to cholesterol, a major component of cellular membranes; the only difference between the two compounds is an extra oxygen molecule on 7-KC compared to cholesterol (Figure 4C). The addition of a cholesterol-like molecule could alter cellular membrane composition, which could block one of the many stages in ZIKV replication that relies on membranes, including entry, genome replication, or budding from the host cell.

### 3.5. 7-Ketocholesterol Dose-Response

**Figure 5:**
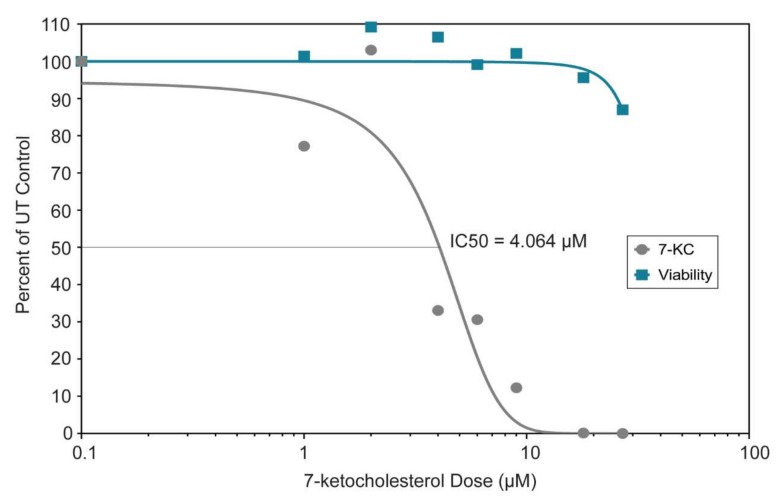
Dose-response curve and viability of Vero cells treated with varying doses (0–27 µM) of 7-ketocholesterol. The curves were generated by GraphPad Prism version 7.04 using the log (inhibitor) vs. normalized response variable slope regression. The regression curve estimates the IC50 at 4.064 μM (R^2^ = 0.9078). The cell viability did not drop below 80% preventing us from determining the EC50.

We generated a dose-response curve to determine the IC50 and range of activity of 7-KC in Vero cells (Figure 5). The IC50 of 7-KC for ZIKV SPH is 4.064 µM, and Vero cells remained more than 80% viable at all 7-KC concentrations tested (0–27 µM). Because of the high cell viability, we were not able to generate a full viability curve over the range of active 7-KC doses, suggesting there is a large range of activity between cell viability and viral inhibition.

### 3.6. 7-Ketocholesterol Time-of-Addition

**Figure 6:**
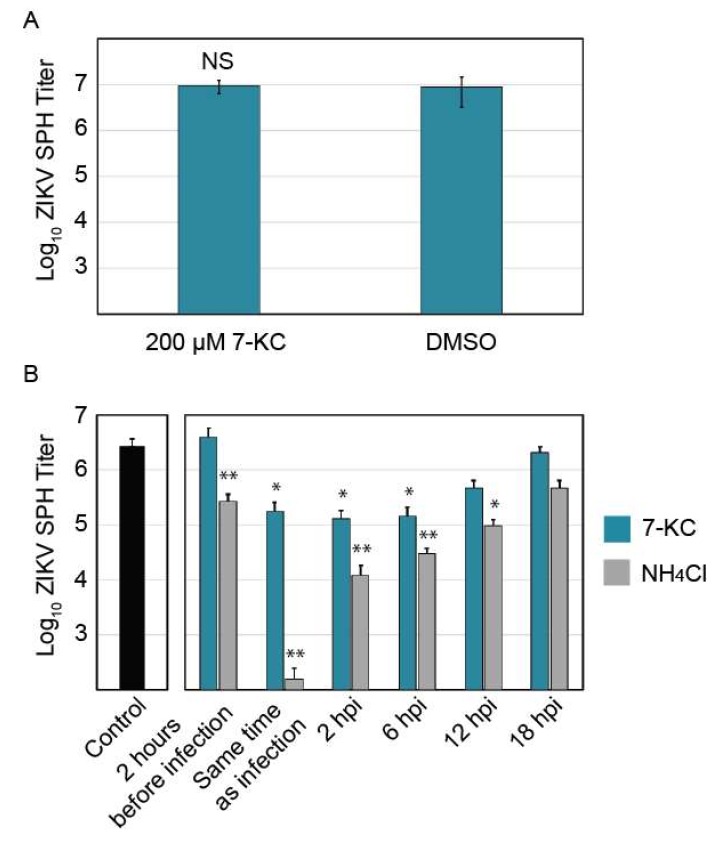
Analysis of 7-ketocholesterol inhibition of ZIKV SPH replication. (**A**) ZIKV SPH preincubation with 200 µM of 7-KC before Vero cell infection. (**B**) 7-KC versus ammonium chloride time-of-addition assay in Vero cells. Error bars represent the SEM. *, *p* < 0.05; **, *p* < 0.01.

To help elucidate the mechanism by which 7-KC inhibits ZIKV replication, we performed a time-of-addition experiment to determine when 7-KC was most effective at inhibiting ZIKV replication. First, we determined if 7-KC directly impacts ZIKV infectivity through a virucidal effect. ZIKV stock was preincubated with a high concentration (200 µM) of 7-KC for 1 h. This mixture was then diluted onto Vero cells so that the 7-KC concentration was below the active range as determined by the dose-response curve (Figure 5). Therefore, any viral inhibition would be caused by 7-KC virucidal effect and not by 7-KC activity within the host cell. As shown in Figure 6A, 7-KC preincubation with ZIKV SPH did not reduce viral titers compared to controls suggesting 7-KC modifies the infected host cell in a way that limits ZIKV replication.

Cells were treated with 7-KC or ammonium chloride 2 h before infection, at the time of infection, and 2, 6, 12, and 18 h following infection. Ammonium chloride raises lysosomal pH, preventing flavivirus entry [28] and was used as an entry inhibitor control. Ammonium chloride treatment 2 h prior to infection and at the time of infection inhibited ZIKV replication, but it was less inhibitory when added later in infection. Interestingly, pre-treating cells with 7-KC 2 h before infection did not reduce viral titers, yet treatment with 7-KC at the time of infection, at 2 hpi, or at 6 hpi all decreased ZIKV titers by 90% (Figure 6B). ZIKV inhibition continued even 12 h after infection, while 18 h after infection only showed a slight decrease in titer. It may take some time for 7-KC treatment to take effect, and adding the drug at 18 hpi (only 6 h before virus was collected) may allow some virions to be produced before the drug was added. These results suggest that 7-KC does not inhibit ZIKV entry and instead inhibits a downstream step in the replication pathways such as viral replication or budding.

### 3.7. 7-Ketocholesterol qRT-PCR

**Figure 7:**
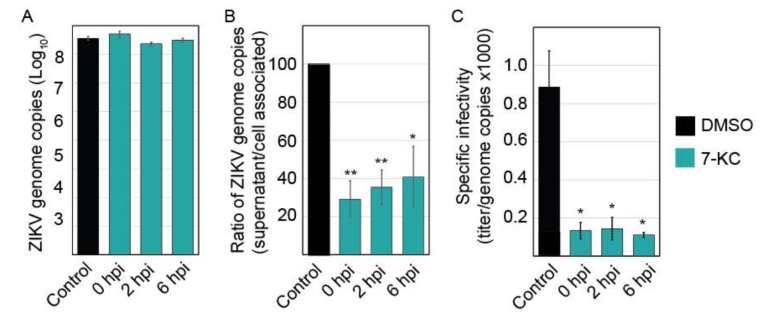
7-KC reduced ZIKV budding efficiency and infectious virion production. (**A**) ZIKV infection was inhibited by adding 7-KC at the indicated time points following infection. Twenty-four hours post infection (hpi), total cellular RNA was harvested and ZIKV genomes were quantified using q-RT-PCR. 7-KC did not significantly alter the level of ZIKV genomes in the cells. (**B**) ZIKV genome copies present in the supernatants were also quantified. To determine the budding/release efficiency we compared the number of genomes present in the supernatant to cell associated genomes and determined the budding efficiency based on DMSO control. (**C**) ZIKV produced in the presence of 7-KC is less infectious than virus grown in DMSO control cells. The specific infectivity of the virions was determined by comparing the titer to the number of genome copies found in the supernatant (×1000). Each RNA sample was run in triplicate during each trial for a total of 3 trials. *, *p* < 0.05; **, *p* < 0.01.

Time-of-addition experiments demonstrate that 7-KC is able to reduce virus production even when added 6 h following infection, suggesting that the compound most likely affects ZIKV production late in the infection cycle. To examine the levels of viral RNA production, we isolated total RNA from infected cells and quantified the number of viral genome copies present. The levels of ZIKV RNA were not significantly altered in the presence of 7-KC (Figure 7A), suggesting 7-KC does not alter RNA production. Although the levels of RNA found in the cells were not significantly different, the number of viral genome copies in the supernatant was reduced, suggesting a budding or release defect. When we compared the number of genomes in the supernatant to the amount in the cells, 7-KC treatment decreases budding/release efficiency to approximately 30% of untreated cells (Figure 7B). While this defect could account for a slight decrease in virion production, it alone could not cause the greater than 90% decrease in infectious virions. Therefore, we hypothesized that the particles budding from the 7-KC treated cells may be less infectious than ZIKV produced from untreated cells. We determined the specific infectivity of the particles by comparing the infectious titer to the number of genome copies in the supernatant samples (Figure 7C). Particles produced in cells treated with 7-KC were significantly less infectious than the DMSO control. Only 1 in approxiamately 9000 genomes were infectious when 7-KC was present compared to 1 in 1200 of control.

## 4. Discussion

To further our understanding of how ZIKV replication interacts with the autophagy pathway, we examined ZIKV replication in the presence of 94 autophagy inducers and inhibitors. Most compounds did not affect ZIKV replication or substantially reduce cell viability, preventing us from assessing the compounds’ effect on replication. When comparing the effects of the compounds in the mammalian and mosquito cell lines, we observed a similar pattern of inhibition for the most part (Figure S2). Some compounds had cell-type specific cytotoxicity which frequently explained any differences in trends. The number of compounds that did not significantly alter ZIKV replication surprised us. While all the compounds in the library have been shown to alter the autophagy pathway, some of the compounds effects may be indirect. Rapamycin, the prototypical autophagy inducer increased ZIKV production in Vero cells, yet slightly decreased production in C6/36 cells. Hydroxychloroquine blocked ZIKV spread in a mouse model [12] and we found it decreased titers in both Vero and C6/36 cells. While trehalose, an autophagy inducer, has been suggested to block ZIKV transmission [29], it was not able to reduce ZIKV titers in Vero or C6/36 cells.

Three compounds, amiodarone-HCl, 7-ketocholesterol, and licochalcone A, inhibited ZIKV replication in Vero cells without reducing cell viability, but neurons only remained viable when treated with 7-KC. 7-KC was also the only compound that reduced ZIKV IbH by more than 90% of control. 7-KC is an oxysterol formed by oxidation of cholesterol [30] and is a major component of oxidized lipoprotein found in atherosclerotic plaque [31]. 7-KC is a biomarker for oxidative stress and elevated levels are observed in blood samples of cancer patients and patients with inflammation [32,33,34]. Although the mechanism remains unclear, sub-toxic levels of 7-KC activates PI3K/mTOR signaling, inducing autophagy [35]. 7-KC has also been linked to enhancing intracellular hydrogen peroxide levels, triggering autophagy [36]. While low concentrations of 7-KC are associated with autophagy induction, high concentrations are cytotoxic and induce apoptosis [36]. Most of the existing research on this compound focuses on it association with atherosclerosis [37], a condition characterized by hardened, plaque-filled arterial walls caused by oxysterol buildup over time [31]. 7-KC also induces inflammation through TLR4 signaling [38]. Currently there is no literature examining 7-KC and antiviral activity against enveloped viruses, however one study examining oxysterols and non-enveloped viruses found 7-KC had no activity against human papillomavirus and human rotavirus with minor activity for human rhinovirus [39].

7-KC will incorporate into cellular lipids and disrupt normal lipid order [40,41], which can impair phagocytosis [42]. A similar oxidized cholesterol-based compound, 25-hydroxycholesterol (25-HC), reduced ZIKV titers in vitro and in vivo by inhibiting viral entry [43]. 25-HC may modify host membranes, preventing efficient fusion [43]. Due to the similar make-up of 25-HC and 7-KC one might predict it would also decrease viral entry. However, our time-of-addition studies suggests 7-KC does not block viral entry or RNA production, but a late stage in viral replication such as viral budding or particle release from the cell. In addition to decreasing the efficiency of particle release, the particles that are released into the supernatant are less infectious.

Interestingly, 7-KC induces autophagy [36], and because ZIKV replication is enhanced by autophagy induction, one could suggest that 7-KC should increase ZIKV titers. 7-KC’s role in inducing autophagy and the antiviral activity we observed may not be related. While 7-KC may increase autophagy flux through activating PI3K/mTOR, its effects on disrupting lipid order may have more profound consequences on ZIKV spread. ZIKV replication, budding, maturation, and release all rely on host lipids and the cellular secretory pathway [44]. Particle infectivity was altered when the infected cells were treated with 7-KC, but the compound had no virucidal effect on intact particles. This suggests that the 7-KC may significantly alter the lipid environment in the organelles that are critical for budding and trafficking of ZIKV.

In conclusion, we identified 7-ketocholesterol as an inhibitor of ZIKV replication in both Vero cells and human neurons. While antiviral, 7-KC only dropped viral titers by an order of magnitude, suggesting the compound would not be clinically relevant. However, we believe that 7-KC will be a useful tool to examine the lipids involved in ZIKV particle production and further explore ZIKV budding and trafficking mechanisms.

## Figures and Tables

**Table 1:** Summary of autophagy compound screen.

# ^a^	Name	Type ^b^	ZIKV SPH Relative Titer in Vero Cells ^c^	Vero Viability ^d^	ZIKV SPH Relative Titer in C6/36 Cells ^c^	C6/36 Viability ^d^
1	Bafilomycin A1	H	0.0 ± 0	55.7 ± 9.5	0.0 ± 0	36 ± 6.1
2	Rapamycin	D	167.9 ± 44.4	66.0 ± 2.6	75.7 ± 47.7	59 ± 7.3
3	Timosaponin A-III	D	0.0 ± 0	38.7 ± 11.9	13.0 ± 5.2	67 ± 3.6
4	3-Methyladenine	H	73.4 ± 4.9	90.7 ± 1.8	155.3 ± 41	120 ± 15.6
5	PI-103	D	42.3 ± 20.6	43.7 ± 6.4	46.2 ± 13.3	39 ± 8.9
6	LY294002	H	264.3 ± 182.9	56.0 ± 6	548.4 ± 221	97 ± 17.2
7	Lithium Chloride	D	85.4 ± 18.7	87.0 ± 1	167.9 ± 68.4	111 ± 10.7
8	L-690,330	D	40.7 ± 10.6	93.7 ± 3.5	66.8 ± 2.6	87 ± 4.6
9	Wortmannin	H	46.9 ± 9	79.0 ± 3.1	190.1 ± 57.8	105 ± 5.5
10	Sodium Valproate	D	110.8 ± 80.4	92.7 ± 1.2	111.4 ± 20.7	111 ± 6.2
11	Verapamil·HCl	D	92.9 ± 20.8	87.7 ± 0.9	89.0 ± 40.8	71 ± 2.9
12	SP600125	H	26.5 ± 15	66.0 ± 2.1	50.3 ± 19.8	132 ± 9
13	Chloroquine	H	109.0 ± 65	89.7 ± 4.4	126.5 ± 44.8	81 ± 17.1
14	Loperamide HCl	D	11.7 ± 3.3	66.7 ± 17.4	0.1 ± 0.1	12 ± 6.1
15	Amiodarone HCl	D	6.3 ± 3.6	84.0 ± 8.2	2.0 ± 0.5	44 ± 15.3
16	Nimodipine	D	32.5 ± 7.3	92.0 ± 1.5	26.9 ± 13.6	41 ± 8.4
17	Nitrendipine	D	66.0 ± 27.1	88.7 ± 1.9	219.7 ± 44.4	62 ± 10.5
18	Niguldipine	D	0.2 ± 0.1	30.0 ± 14.2	0.0 ± 0	5 ± 2.1
19	Penitrem A	D	34.9 ± 10.4	78.3 ± 6.4	29.8 ± 6.4	43 ± 8.1
20	Ionomycin	D	0.0 ± 0	27.7 ± 10.4	0.0 ± 0	1 ± 0.3
21	Rotenone	D	1.8 ± 0.9	61.3 ± 7.7	0.1 ± 0.1	17 ± 5.8
22	TTFA	D	24.4 ± 7.6	90.0 ± 3.5	72.8 ± 9.6	78 ± 19.9
23	Fluspirilene	D	2.5 ± 0.6	56.7 ± 16	1.0 ± 0.7	26 ± 10.3
24	Hydroxychloroquine	H	14.7 ± 7.3	85.3 ± 8.4	25.1 ± 12.6	35 ± 9.7
25	Norclomipramine HCl	H	3.9 ± 0.5	48.7 ± 22.9	13.4 ± 8.7	19 ± 8.9
26	Trifluoperazine·2HCl	D	2.8 ± 0.7	51.0 ± 22.1	0.3 ± 0.3	10 ± 3.8
27	Sorafenib tosylate	D	5.8 ± 1.8	58.3 ± 9.9	9.4 ± 8.8	23 ± 8.8
28	Niclosamide	D	0.1 ± 0	29.7 ± 3.8	0.1 ± 0	2 ± 0.3
29	Rottlerin	D	10.3 ± 5	80.7 ± 8.5	30.3 ± 16.9	40 ± 8.4
30	Caffeine	D	80.6 ± 20.9	104.7 ± 8.6	138.4 ± 37.7	83 ± 4.6
31	Metformin·HCl	D	122.3 ± 41.5	92.3 ± 4.2	75.8 ± 19.2	64 ± 10.3
32	Clonidine·HCl	D	65.4 ± 17.3	97.7 ± 2.6	95.9 ± 53.4	69 ± 5.4
33	Rilmenidine	D	191.2 ± 49.5	97.0 ± 2	161.0 ± 58.1	79 ± 8.3
34	2′,5′-Dideoxyadenosine	D	93.3 ± 31.9	95.0 ± 1.5	317.4 ± 161.9	118 ± 15
35	Suramin·6Na	D	107.4 ± 44.8	92.0 ± 1.2	86.6 ± 23.2	86 ± 8.4
36	(±)Bay K8644	H	34.9 ± 15.2	94.0 ± 3	39.4 ± 10.7	53 ± 11.1
37	Forskolin	H	139.0 ± 32.1	74.7 ± 3.7	302.5 ± 18.3	94 ± 12
38	Pimozide	D	77.9 ± 15.5	90.0 ± 5.7	109.0 ± 22	49 ± 3.6
39	STF-62247	D	45.0 ± 21.4	77.3 ± 11.7	169.8 ± 45.9	49 ± 9.3
40	Spermidine	D	115.0 ± 44.9	78.0 ± 17	90.2 ± 57.4	70 ± 3
41	FK-866	D	7.3 ± 6.5	32.7 ± 2.7	92.7 ± 32.4	48 ± 5.5
42	Tamoxifen citrate	D	3.3 ± 2.3	60.7 ± 18.4	8.2 ± 7.3	32 ± 11.9
43	Minoxidil	D	60.4 ± 27.2	96.3 ± 3.2	145.7 ± 16.6	72 ± 2.6
44	Imiquimod	D	228.5 ± 139.8	91.3 ± 1.2	72.5 ± 18.4	37 ± 8.1
45	Imatinib mesylate	D	91.2 ± 60	81.0 ± 10.7	5.3 ± 2.2	19 ± 5.4
46	AG112	D	77.7 ± 38.2	96.7 ± 3.7	85.5 ± 21.3	80 ± 5.8
47	SU11652	D	0.1 ± 0	1.0 ± 0	0.0 ± 0	1 ± 0
48	Dibutyryl cAMP·Na	H	60.6 ± 36.7	98.3 ± 4.4	142.6 ± 63.9	117 ± 10.7
49	Rolipram	H	103.8 ± 26.8	91.0 ± 3.2	215.4 ± 74.8	127 ± 12.1
50	SB202190	D	13.3 ± 4.2	74.3 ± 5.2	25.9 ± 7.4	44 ± 7.9
51	Brefeldin A	D	5.4 ± 4.9	17.0 ± 2.1	123.5 ± 25	91 ± 8.4
52	Tunicamycin	D	0.5 ± 0.5	56.3 ± 8.7	1.8 ± 0.8	47 ± 7.6
53	Thapsigargin	D	0.4 ± 0.4	38.0 ± 10.5	0.0 ± 0	23 ± 3.9
54	A23187	D	0.5 ± 0.5	17.0 ± 6.7	0.0 ± 0	1 ± 0
55	Capsaicin	D	76.9 ± 29.6	91.7 ± 2.4	48.1 ± 12	66 ± 7.2
56	Dihydrocapsaicin	D	28.7 ± 10.5	92.3 ± 3.8	64.1 ± 16	61 ± 2.2
57	Glucosamine HCl	D	51.8 ± 11.8	99.3 ± 1.9	242.1 ± 45.8	88 ± 5.2
58	DTT	D	60.2 ± 20.1	93.0 ± 1.5	145.7 ± 16.6	79 ± 4.4
59	Deoxycholate·Na	D	76.6 ± 16.6	95.3 ± 2.6	90.6 ± 12.5	83 ± 1
60	8-CPT-cAMP·Na	H	107.8 ± 65.3	92.7 ± 0.7	60.1 ± 18.3	75 ± 3.7
61	EHNA·HCl	H	81.0 ± 7.6	92.3 ± 2	36.4 ± 9.1	55 ± 4.2
62	ABC294640·HCl	D	51.7 ± 29.2	87.7 ± 6.8	26.0 ± 10.8	32 ± 3.5
63	Licochalcone A	D	7.2 ± 2.9	100.7 ± 9.4	51.8 ± 28.5	43 ± 2
64	Curcumin	D	24.1 ± 11.3	75.3 ± 11.3	0.3 ± 0.2	3 ± 0.7
65	Plumbagin	D	0.1 ± 0	2.0 ± 0	21.4 ± 17.5	49 ± 1.5
66	6-Gingerol	D	99.9 ± 63.9	100.0 ± 2.5	109.3 ± 9.3	55 ± 11
67	Akt Inhibitor X·HCl	D	3.5 ± 3.3	32.3 ± 14.2	0.0 ± 0	7 ± 1.9
68	PMSF	D	89.0 ± 7	98.3 ± 2	258.0 ± 145.2	81 ± 6.4
69	MG132	D	0.1 ± 0.1	10.7 ± 2.8	9.5 ± 7.1	31 ± 5.7
70	ALLN	D	123.3 ± 27.3	75.0 ± 17.1	41.6 ± 0.6	49 ± 6.6
71	7-Ketocholesterol	D	7.5 ± 1.3	93.0 ± 4	9.8 ± 3.3	36 ± 5.1
72	SB-216763	H	41.6 ± 30.6	80.0 ± 9	54.0 ± 21.7	63 ± 3.6
73	Tolazamide	H	176.8 ± 50.9	99.0 ± 1.5	120.1 ± 33.7	113 ± 7.3
74	17-AAG	D	36.4 ± 20.8	56. 7 ± 11.8	18.2 ± 11.2	79 ± 16.4
75	Geldanamycin	D	108.4 ± 39.4	61.3 ± 5.7	25.8 ± 14.5	45 ± 5.8
76	C1	D	119.3 ± 34.4	97.3 ± 2.4	101.2 ± 29.5	55 ± 1.7
77	Z36	D	0.1 ± 0.1	0.7 ± 0.3	0.0 ± 0	0 ± 0
78	Rockout	D	169.6 ± 69.3	87.7 ± 3.8	96.2 ± 44.2	76 ± 5.2
79	Go6850	D	1.3 ± 0.9	36.0 ± 9	1.4 ± 1.4	21 ± 0
80	2-Deoxyglucose	D	99.7 ± 15.4	98.7 ± 0.9	108.8 ± 61.2	73 ± 1
81	Etoposide	D	134.0 ± 34.3	84.0 ± 3.6	736.8 ± 89.3	119 ± 12.5
82	SMER28	D	65.0 ± 5.6	92.7 ± 3.5	38.7 ± 12.2	36 ± 5.3
83	Trehalose	D	107.1 ± 16.9	97.7 ± 1.5	245.7 ± 101.3	99 ± 2.9
84	Quinine HCl·2H2O	H	180.2 ± 70.4	89.7 ± 2.7	123.0 ± 28	54 ± 4.6
85	AICAR	H	112.3 ± 32.3	90.0 ± 11.1	252.0 ± 115	125 ± 16.4
86	C2-dihydroceramide	D	36.5 ± 3	101.3 ± 3.7	262.1 ± 51.7	97 ± 5.8
87	Temozolomide	D	89.9 ± 17.5	104.7 ± 1.9	218.7 ± 49.8	113 ± 9.2
88	Resveratrol	D	67.5 ± 13.2	87.3 ± 2.3	154.6 ± 62	81 ± 7.5
89	Staurosporine	D	0.1 ± 0	14.0 ± 1.5	0.0 ± 0	16 ± 5.3
90	PD-98059	H	101.0 ± 23.3	90.7 ± 1.3	197.3 ± 40	93 ± 8.4
91	Anisomycin	H	0.0 ± 0	12.7 ± 4.3	0.0 ± 0	7 ± 0.9
92	Cycloheximide	H	0.0 ± 0	37.3 ± 6.2	0.0 ± 0	24 ± 3.5
93	Pifithrin-µ	H	38.5 ± 12.4	49.0 ± 15	75.3 ± 27.5	72 ± 3.2
94	Nocodazole	H	28.4 ± 23.9	56.0 ± 9.6	182.0 ± 39.5	58 ± 2.9

^a^ Compounds are numbered based on the Enzo library and correlate to Figure 1A and Figure 3A. ^b^ Autophagy inducers are labeled D whereas autophagy inhibitors are labeled H; ^c^ All values are displayed as percent of untreated control ± the standard error of the mean (SEM) from three independent trials. Viral inhibition is color coded as follows: compounds resulting in >90% of control are highlighted in red, between 10–90% are in yellow, and compounds that dropped titers below 10% are in green. ^d^ All values are displayed as percent of untreated control ± the standard error of the mean (SEM) from three independent trials. Cell viability data is color coded as compounds that retained >80% viability are in blue and compounds with <80% viability are in grey.

**Table 2:** Autophagy activity factor.

Compound	Autophagy Activity Factor (AAF)
DMSO	−0.85
Rapamycin	7.74
Amiodarone HCl	60.40
Licochalcone A	12.33
7-Ketocholesterol	27.01

## Supplementary Materials

The following are available online at http://www.mdpi.com/1999-4915/11/1/20/s1; Figure S1: Comparison of autophagy compounds’ effects on ZIKV SPH and IbH; Figure S2: Comparison of the autophagy compounds’ effects on ZIKV SPH in Vero and C6/36 cells.
