# Supplementary material for: The Oxysterol 7-Ketocholesterol Reduces Zika Virus Titers in Vero Cells and Human Neurons
Source: Viruses. 2018 Dec 30;11(1):20. doi: 10.3390/v11010020 (PMC6356585; doi:10.3390/v11010020)
Supplement: Supplementary file 1 [file viruses-11-00020-s001.pdf]

The Oxysterol 7-Ketocholesterol Reduces Zika Virus Titers in Vero Cells and Human Neurons

Katherine A. Willard [1], Christina L. Elling [2], Steven L. Stice [2] and Melinda A. Brindley [3,*]

[1] Department of Infectious Diseases, College of Veterinary Medicine, University of Georgia, Athens, GA 30602, USA; katherine.willard@duke.edu
[2] Department of Animal and Dairy Science, Regenerative Bioscience Center, College of Agriculture and Environmental Science, University of Georgia, Athens, GA 30602, USA; christina.elling@ucdenver.edu (C.L.E.); sstice@uga.edu (S.L.S.)
[3] Department of Infectious Diseases, Department of Population Health, Center for Vaccines and Immunology, College of Veterinary Medicine, University of Georgia, Athens, GA 30602, USA
* Correspondence: mbrindle@uga.edu; Tel.: +1-706-542-5796

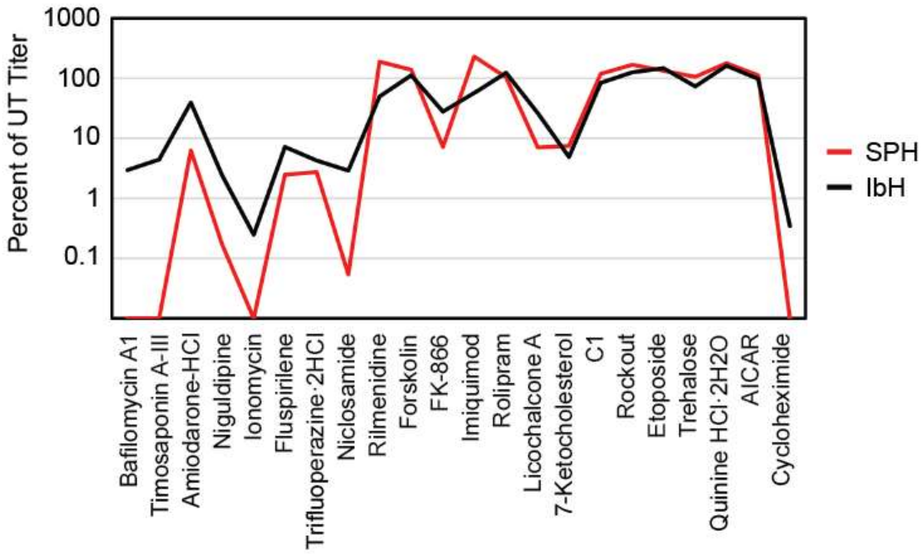

Figure S1. Comparing the effects of select autophagy compounds on ZIKV SPH and IbH virus production. Viral titers were compared to untreated DMSO controls. For the purposes of the comparison, undetectable titers are displayed as 0.01% of control.

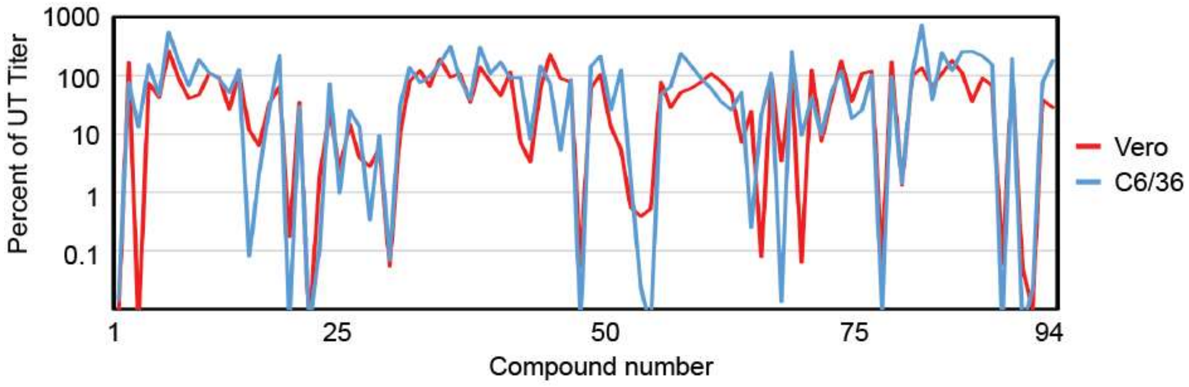

Figure S2. Comparing the effects of select autophagy compounds on ZIKV SPH virus production in Vero and C6/36 cells. Viral titers were compared to untreated DMSO controls. For the purposes of the comparison, undetectable titers are displayed as 0.01% of control.
